# Rethinking the strongest link: VAL, ratings, and team success in Hungarian basketball

**DOI:** 10.3389/fspor.2025.1658676

**Published:** 2025-09-17

**Authors:** Benedek Ágost Nagy, Botond Ágoston Nagy, Ágoston Nagy, József Gáll, Tamás Sterbenz

**Affiliations:** ^1^ School of Doctoral Studies, Hungarian University of Sports Science, Budapest, Hungary; ^2^ Institute of Sport Sciences, University of Debrecen, Debrecen, Hungary; ^3^ Department of Applied Mathematics and Probability Theory, Faculty of Informatics, University of Debrecen, Debrecen, Hungary; ^4^ Sport Economics and Decision Making Research Centre, Hungarian University of Sports Science, Budapest, Hungary

**Keywords:** game analysis, performance, team sport, efficiency, competition

## Abstract

**Introduction:**

This study evaluates the impact of individual player performance–particularly the VAL rating–on team success in professional basketball. It examines whether basketball operates as a “strong-link” sport, where outcomes depend primarily on top-performing players.

**Methods:**

A quantitative analysis was conducted on the 2022/2023 Hungarian NB I/A men's league, using 21 offensive and defensive statistical indicators. Data were collected from official league sources and analyzed using IBM SPSS Statistics version 28.0.

**Results:**

Our findings reveal no significant correlation between individual VAL rankings and team standings. However, offensive and defensive ratings, as well as NET ratings (points scored over opponents per 100 possessions), were strongly associated with team performance, especially among foreign and young players. The VAL metric was more influenced by offensive than defensive performance. Limited playing time and experience may have affected the performance metrics of young players.

**Discussion:**

These results suggest that while basketball reflects strong-link sport characteristics, traditional metrics like VAL may not fully capture a player's contribution to team success. A more comprehensive approach-incorporating both offensive and defensive indicators-could offer a clearer evaluation of player impact. Future research should also explore psychological, tactical, and social factors to better understand individual roles in team performance.

## 1 Introduction

The recent international literature drew our attention to a new method, according to which ball games can be examined from the point of view of how the abilities of individual players influence victory. These studies classify basketball as a strong-link sport, as opposed to soccer, which is considered a weak-link sport (1, 2). This means that in soccer, the ability of the eleventh player largely determines the performance of the team, while in basketball, it is the team's best player(s) who determines the performance of the whole team. Other research also concludes that superstars in basketball increase a team's chance of winning by 8.48 percentage points (3). It is also important to know how many such stars are there in the team and that we use them in balance with teamwork (4). Too much talent can be counterproductive in certain situations (5), especially if players cannot work for one another and together.

In basketball, it is often necessary to evaluate offensive and defensive contributions together to assess a player's overall impact on a team's performance or victory. Determining whether offense or defense is more critical depends on the team's playing style, the country's basketball culture, or even a specific opponent.

Previous research highlights the imbalance in how offense and defense are valued. For example, fans do not necessarily prefer offense over defense in terms of spending. In the NBA, teams compensate offensive production approximately 150% more than defensive production, despite evidence suggesting that win-maximizing teams should value both equally (6). This discrepancy reflects a labor market imbalance in the NBA (7).

In basketball, as opposed to handball, we try to educate and select players during team building who are able to solve complex challenges on both sides of the court. Such players are often called “Two-Way players.” This is crucial in “strong-link” sports like basketball, where key players have to create significant impact on both ends of the court.

According to popular belief, EuroLeague and NBA players create an incredibly large impact on the game. This is partially true when compared with other sports, but then the questions arise: exactly how much influence can a player have on the match and its outcome? Can these players guarantee victory? Can they get a team to the playoffs? By exactly how much will the team's number of wins increase if we sign them? Should we sign a “two-way” player, a really good offensive player, and/or a defensive one?

Even in systems built around a star player, there are possessions where the star is not involved. They might not take the shot, get fouled, or commit a turnover. In addition, every player rests apart from exceptional cases like Wilt Chamberlain's 1962 season, where he played every minute of every game. Desmond Washington, the Hungarian first division's most-used player in 2022/2023, averaged 36 min per game but still missed a significant portion of playtime and possessions. His team averaged 99 possessions per game, and he was on the court in 89.7 possessions. Edwin Devon was the second player who spent the most time on the court, but he was on the bench for more than 15 possessions. His team had 97.6 in average, but he was involved in 81.9 possessions. The top scorer (Perry Darius) of the league spent less time on the court, but thanks to his team's playing style, he was on the court in 84.4 out of his team's 100 possessions.

Star players often dominate offensively, but they cannot do that much in defense. For example, defenders actively participate in approximately only 30% of defensive possessions, while offensive players are involved in 52% of offensive plays. Defensive specialists may have a usage rate below 30%, meaning that they directly impact fewer than one-third of defensive possessions (7). The reason is that good defenders are directly omitted by the opponent from their attacks.

We wanted to find out what makes one more effective link (at team level) stronger than another. What individual statistical indicators can be the ones that contribute to the team's victories. Therefore, we selected those statistical indicators that, according to the literature, have an impact on the team's victory. We wanted to take a look at the individual contribution of the players in these categories, and we wished to compare these indicators with the individual performance of players and the overall effectiveness of the team.

One of the starting points for selecting the variables was Dean Oliver's (2004) four-factor theory. This theory predicted team performance in the NBA with 96%success in recent seasons. According to Oliver (8), the determining factors of performance are effective field goal percentage, turnover percentage, offensive rebounding percentage, and free throw rate.

Other studies also highlighted that field goals and defensive rebounds are among the most critical keys to winning (9–11). Fouls and free throws are also significant, especially in close games (12).

The performance of winning teams depends on the quality of players’ decision-making and well-defined strategic decisions based on the effectiveness and efficiency of field goals in a tactical team environment (13).

Defensive rebounds mean that the team can recover the ball after missed shots by an opponent (13). A successful defensive rebounding team has more opportunities to score points and win matches (9, 11, 14). High-level rebounding performance is closely related to players’ anthropometric characteristics, muscle strength, and technical and tactical preparation (15). As the number of ball possessions and points shot from fast break increases on a team level, the number of high-intensity sprints also increases per player (16).

In recent decades, the offensive game of basketball has changed significantly. One of the main reasons for this is the higher number of three-point attempts and successful three-pointers, while the number of two-pointers (mostly mid-range shots) has decreased (17–19).

This happens for a simple reason: three-pointers are worth more than two-pointers. If someone can shoot 50% from mid-range, this equals only 1 point per possession, whereas achieving the same result requires just 33.3% shooting accuracy from a three-point range (20). Kilcoyne (21) also confirmed the increase in three-pointers and close attempts in his research, as well as the decrease in the number and importance of mid-range attempts. Another driving factor contributing to the switch of spot selection between the long-distance and 16–24 ft is that players are primarily encouraged to drive to the basket or shoot a three-pointer. Kilcoyne found that better teams shoot fewer mid-range shots, maintain a higher pace, and achieve better defensive ratings, which were the most critical factors in winning games during the 2015–2019 NBA seasons.

Multiple factors affect a team's performance during a regular NBA season, for instance, coaches’ tactics, basketball players’ fitness, and game location —home or away— (22). These factors can be divided into two categories, namely, player performance and external factors. The player performance among others includes players’ shooting accuracy, turnovers, rebounding, and free throws. External factors include game locations and coaches’ tactics (23).

Teramoto and Cross (24) discussed the relative importance of performance factors in winning basketball games using 1999–2009 NBA data. Their results indicated that both offensive and defensive field goal percentages are the most critical aspects of the game in the regular season. In addition, efficient offense and defense are essential to success in the regular season.

Among the many performance indicators, the shooting accuracy of field goals in basketball games is found as a critical factor that determines the outcome of basketball games at different levels of professional leagues (25–27). This is true in the regular season as well as in the playoffs (28).

Kilcoyne (21) found a decline in the mid-range jump shot, defined as field goals with a shooting distance in the range of 8–24 ft, by analyzing NBA data from the 2005–2006 season through 2018–2019. The number of field goal attempts and field goal accuracy from each of the four segments in the first quarter is higher than those in the corresponding segment in the remaining three quarters (23).

We are looking for a method that makes the contribution of the players to the victory within the team measurable and that can be compared with other teams. The Hungarian championship is completely unique internationally, because with the introduction of the so-called mandatory youth rule, it is possible to classify players into three categories and examine them accordingly. We intend to obtain scientific results pertaining to the added value of domestically trained, imported, and young players to the team. We aimed to determine the difference between the strongest links and to assess whether the applied test method is sufficient to provide a general answer to this question.

Thanks to the youth rule, not only outstanding foreign and older Hungarian-educated players are needed for a successful season, but also young players who can contribute to victory with their performance, which is based on the previously mentioned theory that basketball is a strong-link sport.

What does this mean? We intend to know whether the players who perform well individually (have the most individual VAL) are really placed in better teams, whether they are more effective, and what are the main differences between these players (from a basketball statistical point of view).

During the research, several questions emerged in our reflections. How many times have the most valuable players in the NBA/the EuroLeague/the Hungarian league won championship titles with their teams in the last 10 years? Is there any association between individual performance and the number of team victories, and the team's overall effectiveness? What are the individual statistical indicators that significantly influence or explain the player's performance in individual ranking? Is there a difference between defensive and offensive indicators? What is the relationship—if any—between individual scoring and position in the individual ranking? What weight do three-pointers carry in this context? To what extent do defensive statistics have an effect on or explain individual ranking? Is it possible to deduce the roles of players in different categories from statistics (point scorers, defenders, three-point shooters, rebounders, etc.)? What are the differences between foreign, domestic, and young players?

## 2 Materials and methods

The data were provided by the InStat (33) video analysis software and Fullcourt, the official basketball statistical program of the Hungarian leagues. In our research, we examined the 2022/2023 regular season of the Hungarian NB I/A group championship. The sample included 210 player profiles. According to the specific rules, we classified these players into three groups:

1. Import group: Players appearing in the league as foreign nationals (68 players).
2. Hungarian-educated group: Players older than the age of 23 competing as Hungarian citizens (60 players) or import players who were competing in the Hungarian leagues and spent 30 months before the age of 21.
3. Hungarian U23 group: Players born in 2000 or later, competing as Hungarian citizens in the given championship year (82 players), referred to as young players hereafter.

From each team, we selected the best player (according to the individual VAL ranks) of the team in each category, the strongest links. We compared a total of three players per team, which amounted to a total of 42 players from 14 teams. However, everyone was analyzed within their own player category, and we worked with a total of 21 variables.

Each player had one full league season in the sample. To obtain more accurate results and avoid bias, we narrowed down the sample. The narrowing criterion was a minimum of 14 matches played (more than 50% of the games), and those who did not meet this criterion were excluded.

As a result, the final sample consisted of 137 players: 39 from the Hungarian U23 group, 43 from the Hungarian-educated group, and 55 from the import group.

### 2.1 Rationale for applying the match participation filter

#### 2.1.1 Representativeness and stability

To assess an entire season, it is important that a player's performance is able to be assessed over a longer period. Players who have played only in a few matches, irrespective of whether they have performed outstandingly or poorly, can distort the averages and the overall statistical picture.

#### 2.1.2 Frequent replacement of foreign players

In the case of imported players, new signings and cuts from the roster during the season are common. A foreign player who has performed well only in three to four games is not really representative of the average performance of the group but could still significantly boost the results if he remained in the sample.

#### 2.1.3 Comparability between groups

The aim of this research is to objectively compare the three groups (foreign, Hungarian-educated, Youth U23). To do this, it must be ensured that each player has contributed to the statistical indicators with a similar volume of appearances; otherwise, the results may be misleading.

#### 2.1.4 Consistency of performance

A player who has played in at least 14 games can be used to gauge how consistent and sustainable their performance has been over the course of a season. This is especially important for metrics such as VAL or individual statistics.

For these reasons, applying this participation threshold was a crucial step in refining the dataset and enhancing the validity of the conclusions drawn from the analysis.

In accordance with the international literature, we used different offensive and defensive statistical indicators. In total, we used 21 individual basketball statistical indictors: individual scored points, layup percentage, mid-range percentage, three-pointer percentage, free throw percentage, defensive rebounds, offensive rebounds, total rebounds, steals, turnovers, personal fouls, assists, blocks, VAL, offensive rating, defensive rating, free throw factor (FTF), assist/turnover percentage, NET rating (points scored over opponents per 100 possessions), efficiency factor, and player's team ranking (Table 1).

**Table 1:** Basketball statistical variables and corresponding notations.

Short basketball glossary	
Variable	Description
HUNL	Hungarian National League
NBA	National Basketball Association
EUL	EuroLeague
REGS	Regular season
PLFF	Playoff (for some leagues, it is called post season)
T.RANK	Team ranking at the end of the regular season
Points (scored)	Scored points (individually by players)
Close two pointer %	Percentage for any shot taken from inside the paint
Mid-range %	Percentage for any shot taken from this area, typically between 10 and 22 ft from the basket
Three point %	Percentage for any shot taken from beyond the three-point line (22 ft)
FT%	Free Throw shooting percentage
Defensive rebounds	Number of defensive rebounds; they occur when a player retrieves the ball after an opponent misses a field goal or free throw attempt
Offensive rebounds	Number of offensive rebounds; they occur when a player retrieves the ball after their own or a teammate's missed field goal or free throw attempt
Total Rebounds	Number of defensive and offensive rebounds combined
Steal	Number of steals. When a defensive player legally takes the ball away from an offensive player (during a pass or dribble)
Turnover	Number of turnovers. When a player loses possession of the ball to the opposing team
Foul	Number of fouls. It is an infraction of the rules, usually involving illegal physical contact with an opponent (shooting foul, blocking foul, charge, etc.)
Assist	Number of assists. When a player makes a pass that directly leads to a score by a teammate
Block	Number of blocks. When a defensive player deflects or stops a shot attempt by an offensive player
VAL	VAL (short for Valorization) is a statistical metric used primarily in European basketball leagues to measure a player's overall efficiency and impact on the game. VAL = Points scored + Rebounds + Steals + Assists + Drawn fouls + Blocks − Turnovers − Missed shots
OffRtg	Offensive rating (by Dean Oliver)—The team's points scored per 100 possessions when the player was on the court
DefRtg	Defensive rating (by Dean Oliver)—The team's points per 100 possessions when the player was on the court
FTF	Free throw factor (by Dean Oliver), made free throw attempts divided by all field goal attempts
A/TO	Ratio number; assists divided by turnovers
NetRtg	The player's overall impact by calculating the difference between OffRtg and DefRtg. It shows how many points a team or player outscores their opponents per 100 possessions
EFF	A player's overall performance and effectiveness in a game, accounting for both their positive and negative contributions

We examined how the most valuable players (MVPs) performed in the Hungarian league in the last 10 years. We also examined the NBA, which is considered the strongest league in the world, as well as the strongest European league, the EuroLeague. We did not choose the MVPs awarded by the leagues, but instead selected the players who were statistically the most valuable. We decided this approach because league MVP awards also take into account team success, media attention, and other circumstances, such as a minimum number of games played. In the Hungarian league, they use value (VAL), in the NBA they use efficiency (EFF) and in the EuroLeague they use performance index rating (PIR). At the same time, the essence of all the three indices is that they include positive and negative individual achievements. This determines who has the most added value.

We examined the relationship of some main variables, such as VAL and Team Ranking and Point scored, and other variables explaining the performance based on the 2022/2023 regular season of the Hungarian NB I/A group championship. For this, we calculated the (linear) correlation coefficient between these variables.

We find it important to note that the measurements are taken from a particular championship, and hence, we cannot claim that our observations are completely free of bias. Hence, although we report the correlation statistics (which can be used in such cases as well as useful descriptive statistics describing the association), we do not report any corresponding inferential statistics such as *P*-values.

For all statistical analyses, we used IBM SPSS Statistics version 28.0.

## 3 Results

### 3.1 The performance of the MVP players in the three championships

MVP players’ team performance in the regular season:

MVP: We selected the MVPs based on basketball statistics rather than league awards, which factor in team success, media attention, and other criteria like team ranking. While different leagues use VAL (Hungary), EFF (NBA), and PIR (EuroLeague), all three indices measure individual contributions by balancing positive and negative performances to determine a player's added value.

In the NBA and EuroLeague, there were 5 seasons where the team of the best-performing individual player also secured the top spot in the regular season, but this never occurred in the Hungarian National League in the last 10 seasons (Table 2).

**Table 2:** The rankings of the best players’ teams in the regular season and playoffs by seasons.

Not every MVP played in the champion team									
Season	Hungarian National League			NBA			EuroLeague		
	Name	REGS	PLFF	Name	REGS	PLFF	Name	REGS	PLFF
13/14	Chism W.	5	4	Kevin D.	2	3–4	Nikola M.	1 (Group)	2
14/15	Hujdurovic N.	11	12	Antony D.	8	8–16	Boban M.	2 (Group)	—
15/16	Ubilla E.	6	7	Stephen C.	1	2	Ioannis B.	3 (Group)	4
16/17	Govens D.	4	2	Russel W.	6	8–16	Nando C.	2	3
17/18	Ivosev T.	9	9	James H.	1	3–4	Luka D.	5	1
18/19	Taylor K	5	3	Giannis A.	1	5–8	Mike J.	12	—
19/20	Carter E.	11	11	Giannis A.	1	5–8	Shane L.	1	2
20/21	Govens D.	3	3	Nikola J.	3	5–8	Nikola M.	1	2
21/22	Persons T	9	8	Nikola J.	6	8–16	Nikola M.	1	3
22/23	Edwin D.	9	10	Nikola J.	1	1	Aleksandar V.	1	2
23/24	Woolridge R.	11	11	Nikola J.	2	5–8	Shane L.	9	—

Data obtained from the 2013–2024 seasons (34–36).

MVP players’ team performance in the playoff/post season:

- NBA: The regular season MVP reached the finals only twice (2015/2016 and 2022/2023). However, it was only in the 2022/2023 season that Nikola Jokić secured the championship title.
- EuroLeague: The top-performing player of the regular season reached the finals five times (2013/2014, 2017/2018, 2019/2020, 2020/2021, and 2022/2023), but only Luka Dončić managed to claim the title in the 2017/2018 season.
- Hungarian National League: The regular season MVP made it to the finals only once, Darrin Govens (2016/2017), but neither did they win the championship nor did they reach the finals in any other season.

### 3.2 Descriptive statistics of player groups

In the study, we examined 21 indicators, of which we present the six most acknowledged ones (Table 3) with their most important descriptive statistics groupwise. These indicators are VAL, OffRtg, DefRtg, NetRtg, Efficiency Factor, and Scored Points.

**Table 3:** Descriptive statistics of player groups including the VAL.

Descriptive statistics of player groups							
Groups	Descriptives	Val	Offensive rating	Defensive rating	Net rating	Points	Efficiency factor
Youth	Mean	104.77	102.77	105.67	−2.90	80.46	87.67
	Max	493.00	310.00	264.00	46.00	334.00	399.00
	Min	11.00	80.00	84.00	−22.00	10.00	3.00
	Median	64.00	94.00	97.00	−5.00	61.00	57.00
	SD	109.92	38.72	34.41	13.05	75.05	92.30
Hungarian	Mean	211.79	98.84	100.63	−1.79	161.12	176.65
	Max	621.00	171.00	201.00	23.00	472.00	520.00
	Min	26.00	83.00	83.00	−33.00	19.00	19.00
	Median	173.00	96.00	97.00	−1.00	142.00	151.00
	SD	146.98	15.88	21.48	12.24	113.65	123.10
Foreigner	Mean	406.31	102.62	103.93	−1.31	306.84	328.29
	Max	700.00	184.00	265.00	23.00	577.00	547.00
	Min	120.00	87.00	83.00	−112.00	106.00	97.00
	Median	424.50	98.00	95.00	3.00	306.00	332.00
	SD	129.32	19.28	34.22	19.96	86.87	105.02

SD, sample standard deviation.

The mean values show clear differences between the three groups of players. The youth players have the lowest VAL (80.46) and points (87.67)—meaning they produce the least measurable performance. The number of both offensive and defensive ratings is approximately 103, but the Net Rating is negative (−2.90), indicating that they concede more points than they score. The Hungarian-educated players display medium performance across all metrics. The VAL is 211.79, average points is 176.65, and Net Rating is close to zero (−1.79). Offensive and defensive ratings are closer to each other (98.84 vs. 100.63), showing a more balanced profile compared with the youth group. The foreign players stand out significantly in all metrics: VAL is 406.31, and the average point is 328.29. The Net Rating is only slightly negative (−1.31), which is surprising given that both offensive and defensive ratings are similar (approximately 103). This may indicate that while they score a lot, they also concede many points in their defensive play.

Among youth players, the standard deviation of VAL and Efficiency Factor is extremely high (109.92 and 92.30), indicating large differences in individual performance. For Hungarian players, the standard deviation of offensive and defensive ratings is the smallest (OffRtg SD = 15.88), meaning that their performance is more stable in these metrics. For foreign players, the standard deviation of Net Rating (19.96) and VAL (129.32) is relatively high, suggesting that the group includes both outstanding and below-average performers.

For youth players, the median VAL (64) is well below the average (80.46). This means a few outstanding performances raise the mean. For Hungarian players, the median and average are closer together, indicating a more balanced performance distribution. For foreign players, the median VAL (424.5) is even higher than the average, suggesting that the majority consistently perform at a high level.

### 3.3 Connection between the groups and the metrics

In what follows, we present the results obtained about the relationship between the metrics (Table 1) and the individual VAL ranking, points (scored), and team ranking between the three groups (foreigners, Hungarian-educated players, and young players).

We apply the following classification based on the correlation coefficient *ρ* obtained:

• Strong Association: 0.75 ≤│*ρ*│.
• Intermediate Association: 0.5 ≤│*ρ*│< 0.75.
• Weak or No Association: │*ρ*│< 0.5.

Note that the sign of the correlation coefficient shows the “direction” of the association; for example, negative values indicate an inverse relationship, where in general, a lower value is associated with a larger performance in the given metric.

### 3.4 Connection with the individual VAL ranking and metrics

**Figure 1:**
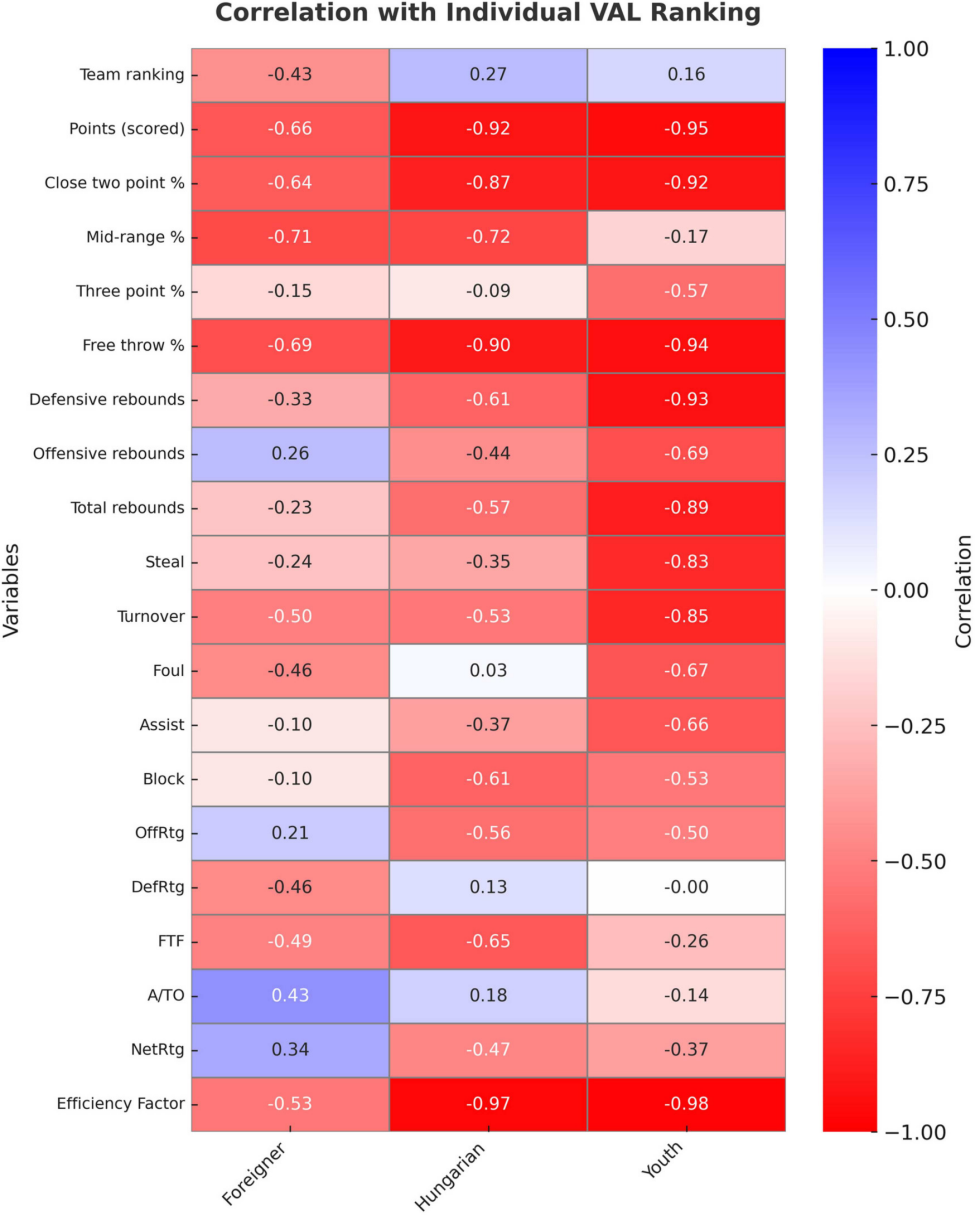
Correlation coefficients between individual VAL ranking and other variables. Data are obtained from the 2022/2023 regular season of the Hungarian NB I/A group championship.

The most expressive indicator in individual evaluation is the individual VAL. VAL = Points scored + Rebounds + Steals + Assists + Drawn fouls + Blocks − Turnovers − Missed shots. We examined its connection with 21 variables. Figure 1 shows the correlation between VAL ranking and other variables.

There is a weak relationship between players’ VAL rankings (the highest value within the team) and their teams’ rankings (the team's place on the table of the league). However, it is worth mentioning that only foreign players have a negative relationship. In other words, as their VAL ranking increased, their team ranking decreased.

There is a negative intermediate association for foreigners with points scored and the two-point shooting percentage, while in the other two groups, there is a very strong negative relationship. For the mid-range percentage, there is a negative intermediate association for foreigners and Hungarian-educated players. There is a weak relationship with the young players. With regard to the three-point shooting percentage, only young players have a negative intermediate relationship. In the other two groups, there is a negative weak relationship. For the free throw percentage, there is a negative intermediate relationship for the foreign players, while there is a very strong negative relationship for the other two groups.

With regard to the defensive rebound, the Hungarian-educated players showed an intermediate association. The young players had a very strong relationship. Only young players showed a strong negative relationship with offensive rebounding. There was a weak negative relationship in the other two groups. For total rebounds, only young players showed a strong negative relationship, for Hungarian-educated players, there was an intermediate relationship, while for foreigners, there was a weak negative relationship.

Only foreign players showed a strong negative relationship with steals, and the other two groups had a weak negative relationship. In terms of blocks, Hungarian-educated and young players showed a negative intermediate relationship. In the DefRtg relationship, neither group showed an intermediate nor a strong relationship.

With regard to assists, only young players showed a negative intermediate association. The assist/turnover ratio (A/TO) did not show an intermediate or strong relationship between any of the groups. In OffRtg, only the Hungarian-educated and young players showed a negative intermediate relationship. In the FTF test, only the Hungarian-educated players showed a negative intermediate association.

In relation to turnovers, foreign and Hungarian-educated players showed a negative intermediate, while young players showed a strong negative relationship. With regard to fouls, only young players showed a strong negative relationship, while the other groups had a weak negative relationship.

In relation to the EFF, foreign players showed a negative intermediate, while the other two groups showed a very strong negative relationship. In terms of NET rating, none of the groups showed an intermediate or strong relationship.

### 3.5 Connection with the individual scored points and metrics

**Figure 2:**
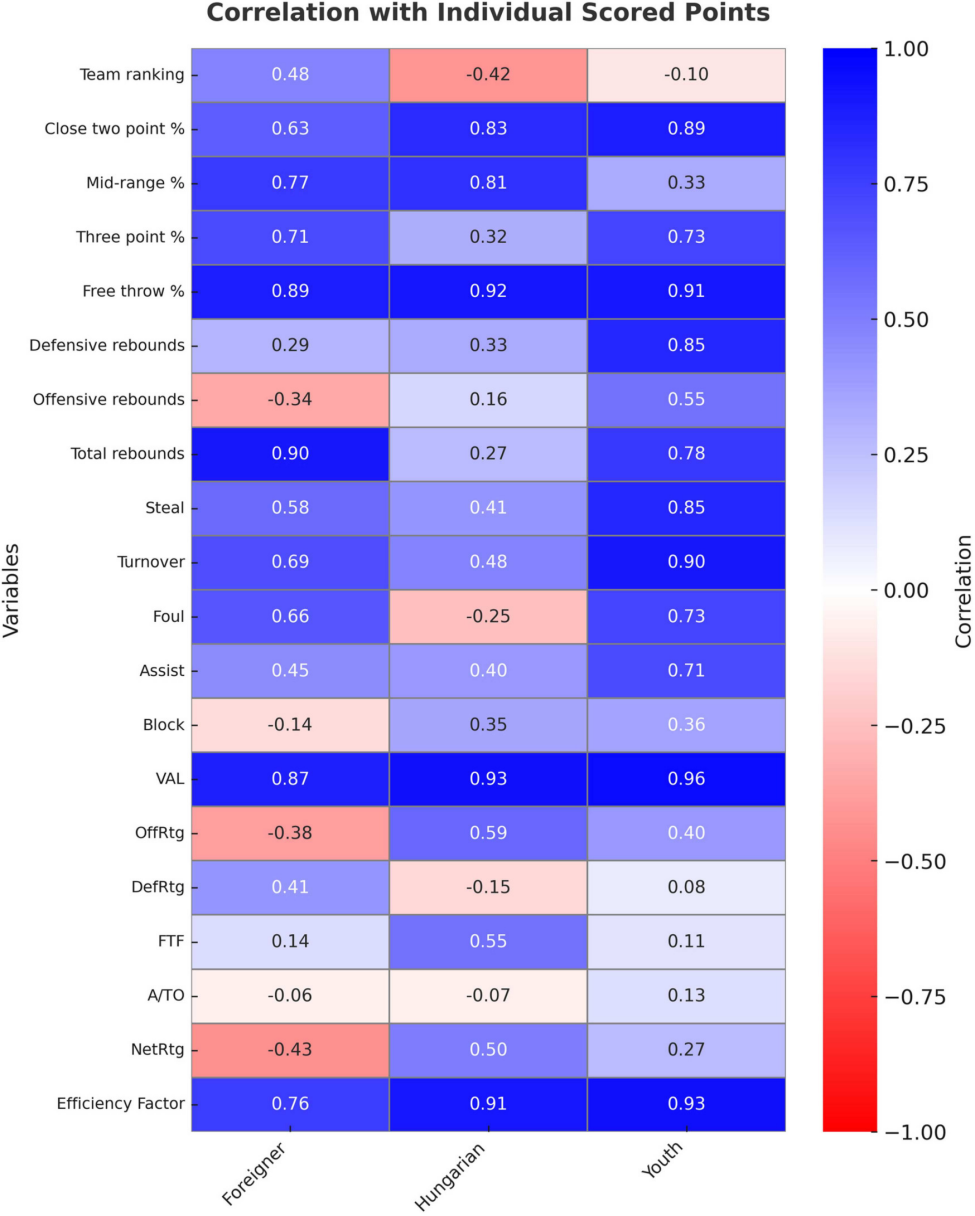
Correlation coefficients between individual scored points and other variables. Data are obtained from the 2022/2023 regular season of the Hungarian NB I/A group championship.

Figure 2 shows the correlation between players’ points (scored) and other variables.

There is a weak relationship between the players’ points scored and the ranking of their teams. However, it is worth mentioning that only foreign players have a positive relationship.

With regard to the two-point shooting percentage, foreign players have an intermediate association. There is a strong relationship in the other two groups. There is a weak relationship with mid-range shooting percentage in young players. In the other two groups, there is a strong relationship. With regard to the three-point shooting percentage, there is an intermediate association among foreigners and young players. There is a strong relationship in all three groups regarding free throw percentage.

Only young players had a strong relationship with defensive rebounding and an intermediate association with the offensive rebound. With regard to the total rebound, both foreign players and young players showed a strong relationship.

Foreign players showed an intermediate connection, while young players showed a strong connection in relation to steals. With regard to block and DefRtg, none of the groups showed an intermediate or strong relationship.

With regard to assists, only young players show an intermediate association. The A/TO did not show an intermediate or strong relationship between any of the groups. In relation to OffRtg and FTF, only Hungarian-educated players showed intermediate association.

In relation to turnovers, foreigners showed an intermediate association, while young players showed a strong association. With regard to fouls, foreigners and young players showed an intermediate association.

All three groups showed a strong relationship with VAL and EFF. Hungarian-educated and young players showed a particularly strong connection. Neither group showed an intermediate or strong relationship regarding NetRtg.

### 3.6 Connection with the team ranking and metrics

**Figure 3:**
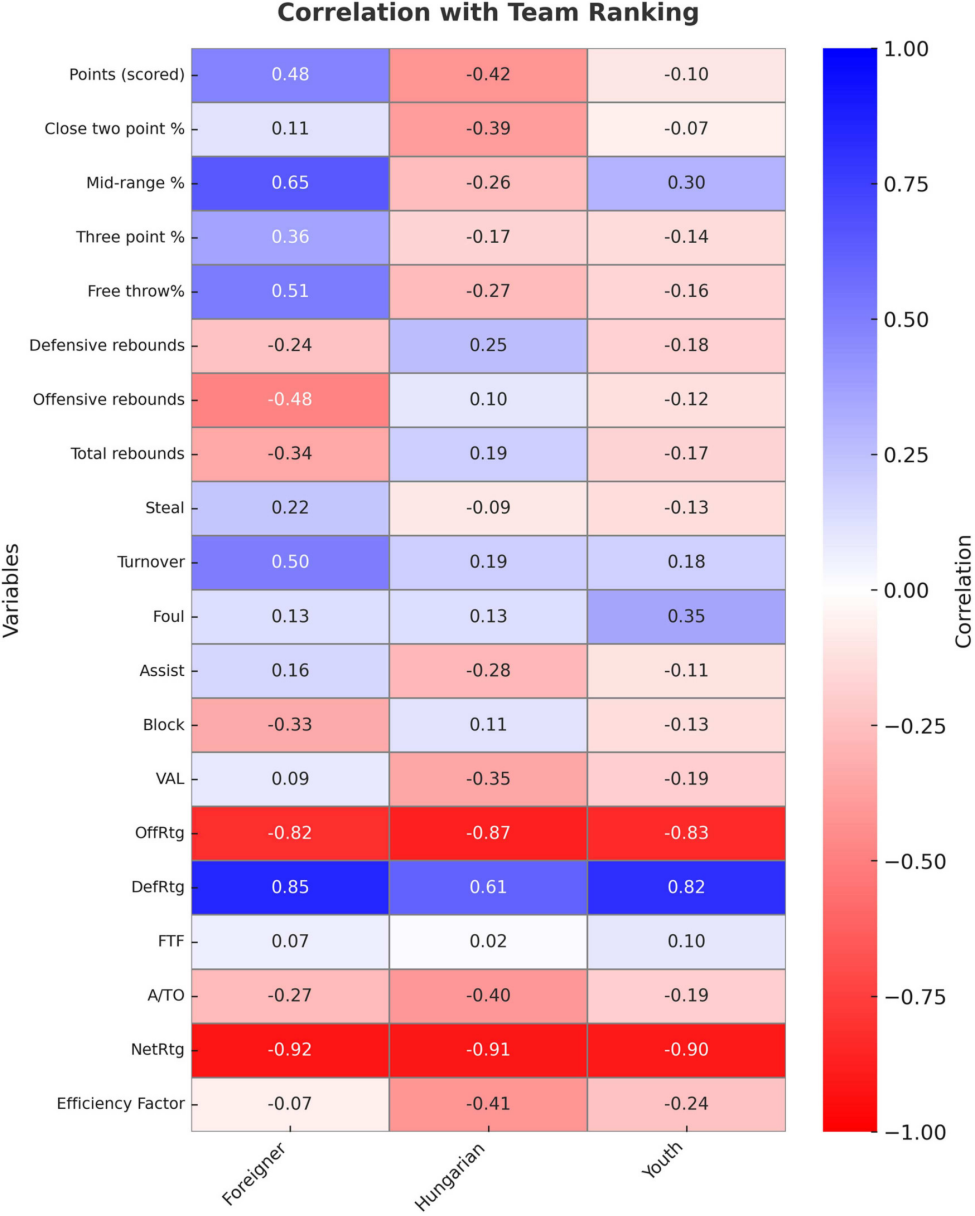
Correlation coefficients between team ranking and other variables. Data are obtained from the 2022/2023 regular season of the Hungarian NB I/A group championship.

Figure 3 shows the impact of indicators on the team's ranking.

None of the groups showed an intermediate or strong relationship with the scored points. Nevertheless, it is worth mentioning that only foreign players had a positive relationship.

They also showed no relationship with the two-point shooting percentage. Only foreign players showed an intermediate association with the mid-range shooting percentage. Neither group showed a relationship with the three-point shooting percentage. Only foreign players showed an intermediate relationship with the free throw shooting percentage.

None of the groups showed a medium or strong relationship in relation to the defensive, offensive, and total rebounds.

Neither group had a connection with steals and blocks. With regard to DefRtg, Hungarian-educated players showed an intermediate association, while foreign and young players showed a strong association.

With regard to assists and assist/turnover ratio and FTF, neither group showed a moderate or strong relationship. For OffRtg, all three groups showed a strong relationship.

With regard to turnovers, only foreign players showed an intermediate connection. Neither group showed a moderate or strong correlation with fouls.

Neither group showed a medium or strong correlation with VAL and EFF. With regard to the NET rating, there was a very strong correlation in all three groups.

## 4 Discussion

Superstar players have their advantages. They increase the team's chances of winning (3). When looking at the impact of superstar players in terms of playoff performance and not just Game Related Statistics (GRS), previous research clearly shows that these exceptional talents can make a significant difference between playoff and non-playoff teams. Such players play a key role in critical situations: they score decisive points from isolation, as a pick and roll handler, post-up player, and catch and shoot situations (29). However, there is also a research that suggests that while points scored from isolation may still be crucial in the playoffs, their frequency is decreasing, while the number of catch and shoot, pick-and-roll-roller, and transition situations is increasing. This suggests that not only the individual solutions of star players play a key role, but also the cooperation within the team and the involvement of other players significantly contribute to success (30). Also, there will be a disadvantage if there are too many superstars in a team, if—for example—their presence disrupts the balance within the team and negatively overturns the hierarchy. Too much talent can be counterproductive in certain situations (5). It should also be acknowledged that even if they are on the court, they cannot influence every ball possession either in attack or in defense (7). Previous research suggests that basketball is a strong-link sport. The stronger is your strongest link, the better you finish in the season (1, 2). However, we found that the strongest link (based on their individual value) in the league had won only one championship in both the NBA and the EuroLeague in the last 10 seasons, but not even once in the Hungarian championship, in the given years. There is generally a moderate connection between MVPs and their team rankings, especially in the NBA and the EuroLeague, where MVPs often play for top-performing teams. In the Hungarian NB I/A league, MVP players are less tied to team rankings.

In the examined sample, there is no strong connection between the player's VAL ranking and the team's position in the regular season in any of the groups. There is an intermediate and strong negative correlation between the VAL ranking and the individual scored points. The relationship is the strongest among young players; the more points they scored, the higher they finished in the individual VAL ranking. The two-point shooting percentage was related to the VAL ranking for all three groups. However, the role of the mid-range shooting percentage was detectable only in foreign and Hungarian-educated players. The three-point shooting percentage was related to VAL ranking only among young players. This reinforces Kilcoyne's (21) claim that the importance and role of mid-range shots is decreasing.

The free throw percentage showed an intermediate and strong relationship in all three categories, meaning that successful free throw shots played an important role in individual performance.

In addition to shooting percentage, defensive rebounding is also very important for winning (9, 14). Nevertheless, only in the case of young players was there an intermediate and strong correlation between individual VAL and field work (in all metrics except shot blocking). There was a particularly strong relationship between defensive rebounds and individual VAL rankings. This is especially valuable from a winning perspective (13).

In none of the groups was there a correlation between the individual points scored by the players and the team's ranking. In terms of individual scoring, the close, mid-range, three-point, and free throw shooting percentages among the imported players had an intermediate and great impact. The number of three-pointers is growing and playing an increasingly dominant role in scoring (18, 21).

In the case of young players, there was no strong connection between the mid-range percentage and individually scored points. However, we found a stronger relationship for the other shot types, which presumably follow international trends in shot selection (17).

The individual defensive and offensive rating values show a strong correlation with the team's position in the regular season for foreign and young players. Hungarian-educated players also had a strong association with OffRtg and an intermediate association with DefRtg. The effective individual defensive and offensive performance of the strong links had a significant impact on their team's championship performance. They played with few mistakes. Therefore, there was a very strong relationship with NetRtg in all three groups. Our research also confirms that it is worth examining the offensive (OffRtg), defensive (DefRtg), and net efficiency (NetRtg) indicators of players separately and not just relying on the VAL that expresses individual performance. In current evaluation systems, individual and team goals are not aligned: individual indicators are often independent of team effectiveness, whereas overall indicators such as IBM do not reliably reflect team performance. These indicators typically measure absolute performance, ignoring efficiency, game pace, and number of attacks. In addition, team-level cooperation—which is essential for success—is not valued, thus allowing room for self-serving, opportunistic play styles. In terms of defense, the current statistical system rewards only spectacular, clear events (e.g., rebounds, steals), while ignoring activities that force opponents to make mistakes—which can distort the assessment of real performance (31).

## 5 Conclusion

There is a performance hierarchy within the groups based on the six main indicators. The foreign players perform on the highest level, then the Hungarian-educated players, followed by the Youth players. In terms of stability, Hungarians are the most balanced, whereas youth players show the most extreme variations. Examining the offensive and defensive rating impact, we can state that all three groups have similar OffRtg and DefRtg values, but the Net Rating is slightly negative across the board. This may indicate that, on average, the league allows slightly more points than it scores, although it could also simply reflect a characteristic of the measurement context.

In the Hungarian NB I/A league, MVP players are less tied to team rankings, likely due to the league's lower competitive balance and the differing roles of players within the teams. When evaluating individual performance, it is not worth simply taking the players’ averages and comparing them. In addition to traditional statistical indicators, we must also examine complex values such as the NET rating or the values derived from ball possessions. Or we should consider standardizing the players for 40 min. This way we can get a more realistic picture. Then, a probability may arise that the best player in the examined season plays for the team at the bottom of the table, while the best player of the champion team is pushed further back in the individual VAL ranking. However, if we were to take the 40-min average of both players, then the best player of the champion team would perform more effectively. This supports the theories mentioned above that the strongest link plays in the best team. This is true only if we start from the assumption that the player's performance does not deteriorate if the number of minutes played is increased.

Because of the tactical skills involved, youngsters take on only close shots and clear open shots from the three-point line. Among young players, the international trend of fading midrange volume is even more pronounced. In addition, the inexperience of young players and the possibility of their being physical challenged can hold them back in some cases. However, these limitations can be consciously addressed and controlled. Young players who follow a structured shooting program improve both their statistical indicators and their competitive performance (32).

Our most important statement is that VAL ranking was more influenced by offensive performance than defensive performance. Neither the individual VAL ranking nor the individual points scored showed a relationship with the team's final ranking; however, the individual defensive and offensive values and the individual NET value did. In other words, good individual performance is more influenced by offensive performance (OffRtg), while defensive performance (DefRtg) is equally important for the team's performance and competitiveness.

An evaluation of the young players shows that their performance is influenced by limited playing time and experience, potentially distorting the results. This study relies solely on quantitative metrics, overlooking psychological, tactical, and social factors affecting performance. We would like to extend the examination to several future seasons and also compare the results with other top European leagues.

## 6 Practical implications

• Coaches should prioritize improving free throw and two-point shooting accuracy to enhance individual player value across all groups.
• Defensive rebounding training should be emphasized, especially for young players, as it strongly contributes to individual and team success.
• Mid-range shooting plays a diminishing role in individual performance, suggesting that teams should focus more on close-range and three-point shooting efficiency.
• Individual offensive and defensive ratings are reliable indicators of team success, especially for foreign and young players, and should guide player development strategies.
• Relying solely on MVP-level players may not guarantee team success, highlighting the need for balanced team composition and collective performance.

## Data Availability

The raw data supporting the conclusions of this article will be made available by the authors without undue reservation.
